# Sero-prevalence of virus neutralizing antibodies for rabies in different groups of dogs following vaccination

**DOI:** 10.1186/s12917-017-1038-z

**Published:** 2017-05-18

**Authors:** R.M.S. Pimburage, M. Gunatilake, O. Wimalaratne, A. Balasuriya, K.A.D.N. Perera

**Affiliations:** 1 Ministry of Health, No.555/5, Elvitigala Mawatha, Colombo 05, Sri Lanka; 2 Department of Physiology, Faculty of Medicine, University of Colombo, No.25, Kynsey Road, Colombo 08, Sri Lanka; 3 Department of Rabies Research and Vaccine Quality Control, Medical Research Institute, Colombo 08, Sri Lanka; 4 Faculty of Medicine, General Sir John Kotelawala Defense University, Kandawala Road, Rathmalana, Sri Lanka

**Keywords:** Antibody, Anti-rabies vaccine, Dogs, RFFIT, Sero-prevalence, Titre

## Abstract

**Background:**

Mass vaccination of dogs is considered fundamental for national rabies control programmes in Sri Lanka, as dog is the main reservoir and transmitter of the disease.

**Methods:**

Dogs were followed to determine the sero-prevalence of antibodies to the rabies virus. Altogether 510 previously vaccinated and unvaccinated dogs with owners (domestic dogs) and dogs without owners (stray dogs) of the local guard dog breed in different age groups recruited from Kalutara District, Sri Lanka. The dogs were vaccinated with a monovalent inactivated vaccine intramuscularly and serum antibody titres on days 0, 30, 180 and 360 were determined by the Rapid Fluorescent Focus Inhibition Test (RFFIT).

**Results:**

The results indicated, a single dose of anti-rabies vaccination fails to generate a protective level of immunity (0.5 IU/ml) which lasts until 1 year in 40.42% of dogs without owners and 57.14% of previously unvaccinated juvenile (age: 3 months to 1 year) dogs with owners. More than one vaccination would help to maintain antibody titres above the protective level in the majority of dogs. The pattern of antibody titre development in annually vaccinated and irregularly vaccinated (not annual) adult dogs with owners is closely similar irrespective of regularity in vaccination. Previously vaccinated animals have higher (2 IU/ml) antibody titres to begin with and have a higher antibody titre on day 360 too. They show a very good antibody titre by day 180. Unvaccinated animals start with low antibody titre and return to low titres by day 360, but have a satisfactory antibody titre by day 180.

**Conclusions:**

A single dose of anti-rabies vaccination is not sufficient for the maintenance of antibody titres for a period of 1 year in puppies, juvenile dogs with owners and in dogs without owners. Maternal antibodies do not provide adequate protection to puppies of previously vaccinated dams and puppies of previously unvaccinated dams. Immunity development after vaccination seems to be closely similar in both the groups of puppies.

## Background

Rabies is a vaccine preventable, zoonotic disease [1]. Still it remains as a serious public health problem all over the world [2]. It is commonly transmitted to other animals and humans through close contact with saliva from infected animals [3]. It is estimated that 60,000 human rabies-related deaths occur worldwide each year [1, 4, 5].

Sri Lanka has invested a large amount of money to control dog rabies. Human deaths due to rabies were further decreased from 56 (2.8 per 1,000,000) in 2007 to 19 (0.05 per 1,000,000) in 2014. This was the lowest incidence reported in Sri Lanka. This reduction was mainly because of mass vaccination of dogs against rabies, mass Animal Birth Control (ABC) programmes, Post Exposure Prophylaxis (PEP) for rabid and rabies suspected animal bites and mass awareness programmes [6, 7]. Being an island once rabies is completely eradicated from Sri Lanka it should be easier to prevent re-introduction of rabies from other countries [7, 8].

The mass vaccination of dogs is considered fundamental in rabies control programmes in many countries including Sri Lanka [7, 9]. The total number of animal rabies cases reported during the 2014 was 746. The majority 81.7% (610) of the animal rabies were dogs. Nearly 50% of the total dog population in the country are vaccinated annually [10]. A strong body of theoretical and empirical evidence indicates that vaccinating 70% of the dog population during annual campaigns should be sufficient to control rabies [11, 12].

According to the vaccine schedule practice in Sri Lanka dogs are vaccinated against rabies at the age of 3 months if the dam is previously vaccinated. If dam is unvaccinated, the 1^st^ vaccine is given to the pup at the age of 6 weeks with a booster at the age of 3 months and subsequently with annual boosters. The study carried out by Gunatilake et al. [13] showed that antibodies transferred from the dam is well below the protective level in the puppies of vaccinated dams at the age of 3 months.

As the government has been investing large amount of money on the treatment of dog bite victims, it is important to determine the ideal time period for the 1^st^ Anti-rabies Vaccination (ARV) for puppies and the level of antibody titres in dogs until the time of revaccination. Measurement of humoral immune response (virus neutralizing antibody titres) in vaccinated dogs is helpful in judging the protectiveness of vaccinated dogs [14]. In a sero-survey carried out in Garborone, Bostwana, it was shown that dogs that had more than one successive vaccination against rabies had better antibody titres than that had received only one vaccination [15]. Another study carried out in Thailand suggested that one dose of tissue culture vaccine in dogs is not adequate to maintain rabies neutralizing antibody titre in serum for 1 year [16].

It is important to know whether dogs in a representative population (specially Sri Lankan local breed) have antibody titres above the protective level (higher than 0.5 IU/ml on WHO recommendation) after the 1^st^ vaccination and whether the antibody titres are maintained above the protective level until the time of annual revaccination [17].

This study was carried out to determine the pattern of immunogenicity and the suitable time for booster vaccination among different age groups of dogs without owner and owned dogs, in order to make recommendations regarding dog vaccination for rabies control in Sri Lanka.

## Methods

### Study design and sample recruitment

This study was carried out in Kalutara district, Sri Lanka. It has 11 MOH (Medical Officer of Health) areas and consists of multi cultural, rural and urban human population. Out of nearly 2000 temporary vaccination centers distributed in 11 MOH areas in the district 37 temporary vaccination centers were randomly selected for the study. In 2014 the average number of confirmed rabies cases in animals throughout the district was 7 per year. Out of 19 total human deaths reported in Srilanka in 2014, one death was in Kalutara district [7].

**Fig. 1:**
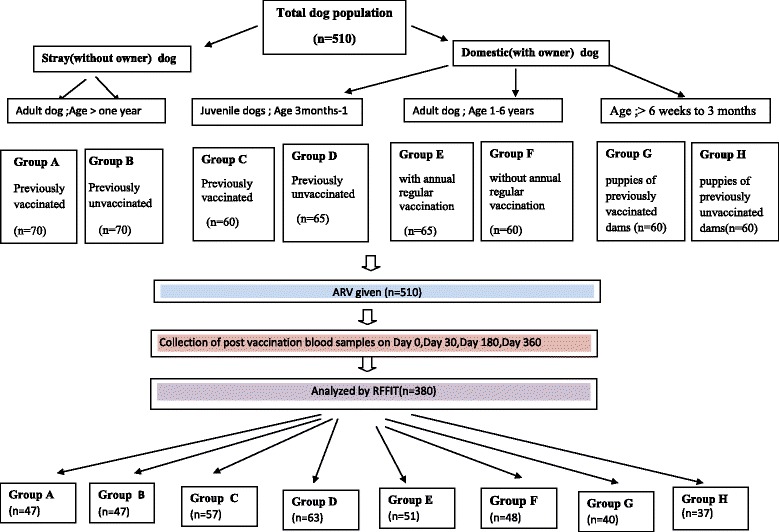
Study Population

A total of 510 apparently healthy dogs with owners and without owners of the local breed were recruited based on pre-defined age groups and previous vaccination history (Fig. 1 and Table 1). Previously vaccinated groups contain dogs with at least one vaccination with ARV.

**Table 1:** The groups included in the study under pre-defined criteria and the number of animals recruited for each group initially

Group		Age	Vaccination history against rabies	No. of Dogs recruited
A	Stray (dogs without owner)	> one year	With previous vaccination	70
B			Without previous vaccination	70
C	Domestic (dog with owners)	3 months −1 year	With previous vaccination	60
D			Without previous vaccination	65
E		>1to 6 years	With regular annual vaccination	65
F			Without regular annual vaccination	60
G		> 6 weeks to 3 months	Puppies of vaccinated dams	60
H			Puppies of unvaccinated dams	60
Total				510

To recruit dogs with owners, a message was sent through dog handlers from house to house requesting them to bring their dogs for vaccination with the vaccination card as evidence for previous vaccination. Dogs were recruited after general clinical examination, that was carried out by a veterinarian. Pedigreed dogs, pregnant or sick animals (e.g. with anorexia, pallor, large wounds), dogs with a known allergy to rabies vaccine were excluded. Vaccination history of domestic animals was noted down from their previous vaccination records.

Some areas in Kalutara district, which were not covered by the government anti-rabies programme were selected for the recruitment of unvaccinated dogs without owners. Previously vaccinated dogs without owners were identified by the presence of tattooing mark and/or red dog collar with a confirmation from villagers from the areas covered by the anti-rabies programme. The dogs without owners were caught and restrained by specially trained skilled dog catchers. A special tattooing number and a photograph were used for easy identification of dogs without owners for the follow up.

### Anti-rabies vaccine and vaccination

A monovalent chemically inactivated vaccine (Nobivac® Rabies) recommended by the WHO for rabies control programmes was used to vaccinate all groups of dogs [7, 18]. This imported vaccine contained >1.0 IU antigenic content per 1 ml dose and the virus was grown on the Baby Hamster Kidney-21 (BHK-21) connective tissue cell line. As recommended by the manufacturer a single dose (1 ml) of vaccine was injected intramuscularly on day 0 (D0). After vaccination, the dogs without owners were released to their territories.

### Collection of blood samples

A venous blood sample (2–3 ml) was collected from the cephalic vein of each animal on day 0 (D0) before vaccination and on day 30 (D30), 180 (D180), 360 (D360) post vaccination. Samples were kept for 1 h at room temperature for separation of serum and transported in ice to the Medical Research Institute (MRI), Colombo. Serum was separated and stored at ^−^70 °C until analyzed by Rapid Fluorescent Focus Inhibition Test (RFFIT) to determine the titres of virus neutralizing antibodies [19].

### Analysis of antibody titres

The antibody titres were analyzed using the Rapid Fluorescent Focus Inhibition Test (RFFIT). Rapid Fluorescent Focus Inhibition Test (RFFIT) is a tissue culture virus neutralization assay, which measures the level of protection against rabies in humans and animals and consider as the “Gold standard method”. The assay measures the amount of antibodies present in serum that neutralizes and block rabies virus from infecting the cells [19]. Infected cells were then identified by fluorescent microscopy. The endpoint titer was calculated using Reed & Muench formula [20]. Serum of each animal was analyzed in the same run to minimize variation.

### Statistical analysis

Antibody titres of dogs in different groups were presented as mean, median and inter quartile range (IQR). An antibody titer of 0.5 IU/ml was considered as the protective level [7, 8, 21] and the percentage of protected dogs in each category was calculated.

Difference between means in different groups according to age and vaccination status was calculated and compared. Multivariate analysis was applied to compare antibody titres and significance was considered at 0.05 level. Statistical analysis was done using a computer software package – SPSS version 10.

## Results

Of the total recruited 510, 428 dogs were available in the follow up until D360 due to unavailability of recruited dogs. However 380 blood samples were available for analysis of antibody titres as some of the samples were haemolyzed.

**Fig. 2:**
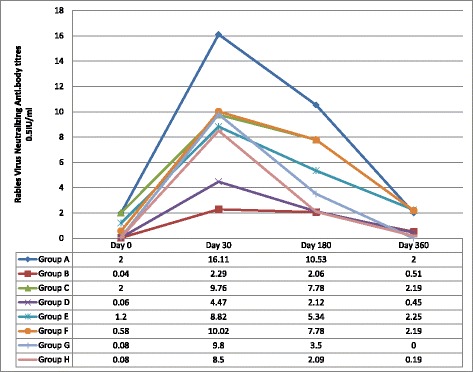
Comparison of median antibody titres of all groups (A-H) without outliers. (Group A- Stray dogs with previous vaccination history; Age > one year, Group B- Stray dogs without previous vaccination history; Age > one year, Group C- Domestic juvenile dogs with a previous vaccination history: Age 3 months – 1 year, Group D- Domestic juvenile dogs without a previous vaccination history: Age 3 months – 1 year, Group E- Adult domestic dogs with regular annual vaccination history :Age 1 to 6 years, Group F- Adult domestic (dogs with owner) dogs without regular annual vaccination history: Age 1 to 6 years, Group G- Puppies of vaccinated domestic dams: Age > 6 weeks to 3 months, Group H- Puppies of unvaccinated domestic dams: Age > 6 weeks to 3 months)

**Fig. 3:**
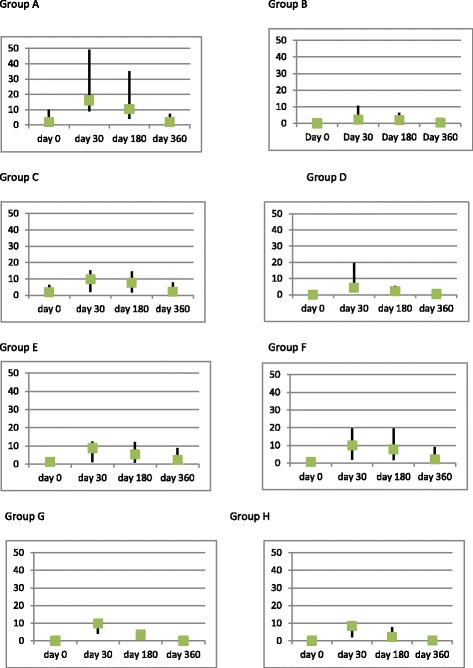
Median antibody titers of all groups A-H ﻿with error bars. Green boxes represent medians, error bars range from 25th quartile to 75th quartile. **(Group A**- Stray dogs with previous vaccination history;Age > one year, **Group B**- Stray dogs without previous vaccination history; Age > one year, **Group C**- Domestic juveniles dogs with a previous vaccination history: Age 3 months – 1 year, **Group D**- Domestic juveniles dogs without a previous vaccination history: Age 3 months – 1 year, **Group E**- Adult domestic dogs with regular an nual vaccination history: Age 1 to 6 years, **Group F**- Adult domestic dogs without regular an nual vaccination history: Age 1 to 6 years, **Group G-** Puppies of vaccinated domestic dams: Age > 6 weeks to 3 months, **Group H**- Puppies of unvaccinated domestic dams: Age > 6 weeks to 3 months)

The mean, 95% confidance intervals, median antibody titres, Inter Quartile Ranges (IQRs) are shown in Table 2. The comparision of protective levels (0.5 IU/ml) of rabies virus neutralization antibody titres of dogs in different age groups on D0, D30, D180 and D360 are shown in Tables 3, 4, 5 and 6. Comparison of median antibody titres of dogs in all groups is graphically presented in Fig. 2, with the removal of outliers. Median antibody titres of all groups with error bars is presented in Fig. 3.

**Table 2:** Comparison of mean, 95% confidence intervals, median, Inter Quartile Range in groups A to H

Group	Days	Mean	Median	Minimum	Maximum	IQR	% with protective titer
Group A (*n* = 47) Previously vaccinated Adult stray dogs	0	6.66 (4.05–9.92)	2 (1.32–8.74)	0.02	49.09	9.4	70.21
	30	51.85(35.90–71.27)	36.44 (13.74–49.05)	1.96	269.76	41.1	100
	180	22.89 (15.11–31.20)	10.53 (8.43–14.32)	0.4	100.4	31.17	100
	360	7.177 (4.25–10.88)	2 (1.62–4.01)	0.19	49.09	8.08	82.98
Group B (*n* = 47) Previously unvaccinated Adult stray dogs	0	0.13 (.08–.19)	0.65 (0.2–.08)	0.02	0.8	0.08	6.4
	30	12.61 (7.23–19.89)	4 (2.10–9.80)	0.02	102.6	11.68	87.24
	180	9.16 (4.74–15.29)	2.15 (1.78–5.48)	0.02	110.2	7.75	87.24
	360	3.89 (1.93)	0.67 (47–1.70)	0.02	50.24	1.85	59.82
Group C (*n* = 47) Previously vaccinated domestic juveniles	0	15.99 (8.06–26.10)	2.7 (1.63–6.78)	0.03	177.5	8.53	78.72
	30	34.77 (18.07–56.20)	10.74 (5.40–11.59)	0.03	269.79	25.11	95.74
	180	27.09 (13.40–45.05)	8.34 (2.90–13-84)	0.06	269.83	17.76	93.62
	360	21.59 (9.00–37.74)	2.90 (1.98–7.78)	0.08	229	8.31	78.72
Group D (*n* = 63) Previously unvaccinated domestic juveniles	0	0.11 (0.07–0.14)	0.06 (0.02–0.08)	0.02	0.8	0.07	1.59
	30	17.39 (11.34–24.13)	9.40 (4.07–10.74)	0.02	125	17.44	93.65
	180	11.21 (6.05–17.26)	2.50 (2.12–5.20)	0.02	124.02	6.34	93.65
	360	3.04 (1.53–4.71)	0.44 (0.28–0.52)	0.02	27.4	1.7	42.86
Group E (*n* = 51) Adult dogs with regular annual vaccination	0	13.62 (6.01–24-24)	29(.56–2.96)	0.03	214.63	7.28	76.47
	30	29.81 (14.55–46.80)	10.02 (2.50–11.04)	0.03	269.79	18.87	96
	180	39.47 (20.48–60.20)	10.50 (2.35–12.46)	0.06	269.83	18.03	88
	360	24.23 (11.11–40.90)	2.56 (2.19–8.93)	0.36	229	10.21	78
Group F (*n* = 48) Adult dogs without regular vaccination	0	6.84 (3.14–11.06)	0.93 (0.55–2.28)	0.03	51.41	5.6	77.08
	30	25.73 (15.20–40.34)	10.74 (4.30–19.54)	0.72	269.76	17.54	100
	180	16.95 (11.12–23.80)	9.41 (2.35–15.56)	0.06	96.5	17.85	95.84
	360	9.69 (5.91–13.97)	2.95 (2.07–8.93)	0.26	47.98	9.15	83.33
Group G (*n* = 40) Puppies of previously vaccinated dams	0	0.10 (0.07–0.14)	0.08 (0.08–0.08)	0.02	0.44	0.03	0
	30	10.66 (7.93–13.74)	9.80 (9.70–10.20)	0.4	49.09	6.3	97.5
	180	4.63 (3.16–6.46)	3.31 (2.00–5.35)	0.04	32.5	5.64	82.5
	360	0.23 (0.15–0.35)	0.09 (0.08–0.19)	0.04	1.8	0.32	7.5
Group H (*n* = 37) Puppies of previously unvaccinated dams	0	0.07 (0.06–0.09)	0.08 (0.04–0.08)	0.02	0.38	0.04	0
	30	12.56 (8.32–17.15)	8.50 (4.70–10.45)	0.08	51.45	9.75	94.59
	180	4.76 (3.00–6.80)	2.09 (0.59–5.40)	0.08	25.6	7.31	78.38
	360	0.32 (0.21–0.44)	0.19 (0.09–0.40)	0.04	1.5	0.36	10.81

**Table 3:** Comparison of protective levels and percentage of rabies virus neutralization antibody (different age groups A to B) titers in previously vaccinated and unvaccinated adult stray dogs

Day	Group A		Group B		*P* value
	Stray (ownerless) dogs with previous vaccination history;Age > one year (*n* = 47)		Stray (ownerless) dogs without previous vaccination history;Age > one year (*n* = 47)		
	No. with antibody titre ≥0.5 IU/ml	% with antibody titre ≥0.5 IU/ml	No. with antibody titre ≥0.5 IU/ml	% with antibody titre ≥0.5 IU/ml	
0	33	70.21	3	6.4	0.000*
30	47	100	41	87.23	0.026*
180	47	100	41	87.23	0.059
360	39	82.98	28	59.57	0.041*

*Indicates the significance level at 0.05 when groups A (Stray dogs with previous vaccination history) & B (Stray dogs without previous vaccination history) are compared

**Table 4:** Comparision of protective levels and percentage of rabies virus neutralization antibody (different age groups C to D) titers in previously vaccinated and unvaccinated domestic juvenile dogs

Day	Group C		Group D		*P* value
	Domestic juvenile dogs with a previous vaccination history: Age 3 months – 1 year, (*n* = 47)		Domestic juvenile dogs without a previous vaccination history: Age 3 months – 1 year (*n* = 63)		
	No. with antibody titre ≥0.5 IU/ml	% with antibody titre ≥0.5 IU/ml	No. with antibody titre ≥0.5 IU/ml	% with antibody titre ≥0.5 IU/ml	
0	37	78.72	1	1.59	0.000*
30	45	95.74	59	93.65	0.487
180	44	93.62	59	93.65	0.643
360	37	78.72	27	42.86	0.000*

*Indicates the significance level at 0.05 when groups C (domestic juvenile dogs with a previous vaccination history) & D (domestic juvenile dogs without a previous vaccination history) are compared

**Table 5:** Comparision of protective levels and percentage of rabies virus neutralization antibody (different age groups E to F) titers in regular annual and without regular annual vaccinated adult domestic dogs

Day	Group E		Group F		*P* value
	Adult domestic dogs with regular annual vaccination history: Age 1 to 6 years (*n* = 51)		Adult domestic dogs without regular annual vaccination history: Age 1 to 6 years (*n* = 48)		
	No. with antibody titre ≥0.5 IU/ml	% with antibody titre ≥0.5 IU/ml	No. with antibody titre ≥0.5 IU/ml	% with antibody titre ≥0.5 IU/ml	
0	39	76.47	37	77.08	0.566
30	49	96	48	100	0.133
180	45	88.23	46	95.84	0.095
360	40	78.43	40	83.33	0.275

**Table 6:** Comparision of protective levels and the percentage of rabies virus neutralization antibody (different age groups G to H) titers in puppies of previously vaccinated and unvaccinated dams

Day	Group G		Group H		*P* value
	Puppies of vaccinated domestic dams: Age > 6 weeks to 3 months (*n* = 40)		Puppies of unvaccinated domestic dams:Age > 6 weeks to 3 months (*n* = 37)		
	No. with antibody titre ≥0.5 IU/ml	% with antibody titre ≥0.5 IU/ml	No. with antibody titre ≥0.5 IU/ml	% with antibody titre ≥0.5 IU/ml	
0	0	0	0	0	
30	39	97.5	35	94.59	0.470
180	33	82.5	29	78.38	0.433
360	3	7.5	4	10.81	0.203

Dogs in the previously vaccinated groups had higher (2 IU/ml) antibody levels to begin with and had a higher antibody level on day 360 than the unvaccinated groups. They showed a very good antibody level at day 180. Unvaccinated animals started with low antibody levels and returned to low levels by day 360, but had a satisfactory antibody level by day 180.

As per Table 2, the antibody titres in puppies of both vaccinated and unvaccinated dams were very low on day 0. Irrespective of the presence of maternal antibodies, titres in puppies after the first vaccination, came only to very low levels by day 360. Data in group G (previously vaccinated dams) indicates that puppies in that group do not have protection by maternal antibodies until the time of primer vaccination.

## Discussion

This is the first comparative study conducted to investigate virus neutralizing antibody titre development (humoral immunity) in stray and domestic dogs and in puppies of vaccinated and unvaccinated dams after an anti-rabies vaccination compared to the antibody titre on the day of vaccination. Previous studies indicate that peak titre of antibodies is generally reached between 4 to 6 weeks after vaccination if the antigenic response is stimulated for the first time [22]. If it is a subsequent stimulation, the time taken for the development of peak titres should be less than this [23]. As per the results of groups A to F, animals with previous vaccination history have antibody titres above the protective level (> 0.5 IU/ml) on D0 and have a higher antibody titre on D360 too (Table 2). Animals without previous vaccination history start with low antibody titres and return to low titres by D360, but have a satisfactory antibody titre at D180. Antibody titre development observed in domestic dogs and puppies in the preliminary study conducted in Kotte, Sri Lanka by Gunatilake et al. [13] was almost similar to the present study. A study done by Seghaier et al. [24] in Tunisia, showed a similar pattern when serum samples of vaccinated dogs against rabies were analyzed. Similarly, Adeyemi et al. [25] compared single vaccination and booster coverage and noted that booster vaccination developed significantly higher level of antibodies. Bunn [26] and Sage et al. [27] reported that single injection of rabies vaccine did not produce a long lasting protective level of antibody titre in a significant number of animals.

Antibody titres on D30 of 5 dogs in group A were between 269 IU/mL to 123 IU/mL which resulted an unusual peak in the median value and therefore to minimize this deviation, these outliers were removed during analysis. This deviation in the antibody titres in individual dogs specially in the stray population could have been resulted due to various reasons such as interval between vaccinations, health or nutrition status, sex and age of the animal (1 to 6 years) and sub-clinical infections. We conducted this study in their natural environment without any intervention which mimics the similar situation in mass vaccination programmes. Also, it was not an objective of our study to find out reasons for individual variation in antibody titres following vaccination.

Antibody titres of dogs and puppies on D30 in all groups except dogs in group A show that 8.4% of animals have not achieved protective levels of titres. The lack of proper protective antibody level after the vaccination of dogs against rabies was reported by Tepsumathanon et al. [29] and Sage et al. [27]. In contrast, Chomel et al. [28] found that 97% of the animals had titres above 0.5 IU/ml D360 after vaccination.

When considering the number of animals in group D (previously vaccinated), only 42.7% of animals had protective antibody titres at D360. Authorities should pay more attention during vaccination programmes to animals in group D which included juvenile dogs without previous vaccination history; aged 3 months-1 year and who are more active and have closer contacts with humans. They carry the greatest risk similar to the puppies in groups G and H. Of the human rabies cases in Thailand, 57% were bitten by puppies under 3 months of age [29]. More than 50% of dogs in group D and more than 80% of puppies in groups G and H did not have antibody titres on D360. Kasempimolporn et al. [22] analyzed serum antibody titres to rabies virus in 32 puppies before primary vaccination and only five showed protective level of antibody [30]. Jakel et al. [31] reported that low levels of neutralizing antibodies (less than 0.5 IU/ml) did not protect the animals. Therefore, it is necessary to give two anti-rabies vaccines to animals in groups D, G and H at a suitable interval with annual boosters. The recommendation of Adeyemi et al. [23], is to give 2 vaccines 1–3 months apart with annual boosters after that to all newly acquired pet dogs and cats in canine endemic regions [25].

Although the dogs in groups B and D were without previous vaccination history, the development and maintenance of antibody titres above the recommended protective level could be acquired by a primary vaccination in majority of dogs until D180. This is an indirect evidence for the efficacy of the vaccine used in the study. However, 40.42% and 57.14% dogs in groups B and D out of 47 and 63 recruited for those groups did not have protective level of antibody titres by D360. According to Cliquet et al. [24] the number of dogs who had antibody titres below the protective level was 7.8% after two months, 19.1% between 2 and 4 months, 25% between 4 to 6 months, 22% more than 6 months after the primo-vaccination in 1351 dogs [32]. The very low humoral response observed in the present study on D0 in unvaccinated dogs could be a technical issue with the RFFIT as it compares antibody titre with a reference sera sample.

However, we observed a large individual variation in the humoral response in animals irrespective of previous vaccination (e.g. *minimum* and maximum titres were between 1.96 and 269.76 IU/ml compared to 0.02 and 102.6 IU/ml on D30 of dogs in groups A and B respectively). Similar results were observed by Cliquet et al. [24] in the study carried out using 25,000 sera samples of dogs and cats vaccinated against rabies [32]. We used a monovalent anti-rabies vaccine recommended by WHO and used by the Government Rabies Control Programme for many years. After purchasing from the local supplier vaccine was stored under recommended conditions of the manufacturer until it is used. Therefore, type and efficacy of vaccine could not be a major cause for the variation in the humoral response that we observed as there is a good response by the animals in certain groups for the vaccination. We made every effort to recruit healthy dogs after a general examination of the animal which is the accepted method in mass vaccinations. It is difficult to comment about the physical status of the animal by the general examination if it is not very obvious. Therefore, whether the discrepancy observed in the humoral response in dogs in different groups is due to the nutritional status of individual animals or not cannot be ruled out. When grouping animals we considered similar age categories; even with this categorization we observed discrepancies in the humoral response. However, Cliquet et al., [24] had observed a significant difference in the humoral response in dogs between primo-vaccinated and multi-vaccinated groups while it was not significant among similar groups of cats [32]. The method we used for the determination of humoral response is RFFIT which has a higher sensitivity recommended by the WHO and well established at the MRI. Therefore, sensitivity of RFFIT is not a factor for the differences observed in the humoral response among animals for the anti-rabies vaccination.

Humoral response induced by vaccination has been identified as an important method of control and prevention of rabies which is a deadly zoonotic disease. Considering the cut off value of 0.5 IU/ml for antibody titres as recommended by the WHO for human sera, our results show that a single vaccination to dogs without owner and owned dogs without a previous vaccination history and to puppies fails to produce an adequate immune response on D360 in many dogs. There were animals in other groups with past vaccination history who did not have adequate protection. Similar patterns of data had been observed by research groups in other countries as well [33–35]. However, Barth et al., indicates by analyzing many experimental results that dogs have a very high probability of survival after a later rabies infection, even if serum does not contain detectable anti-rabies antibodies irrespective of the period of time elapsed since the vaccination [18].

Without measuring immunogenecity (both humoral and cell mediated) in an animal following vaccination and without doing challenge studies it is difficult to consider whether an animal is well protected or not. Irrespective of this hypothesis, based on the results of our study, it is necessary to vaccinate puppies at an early stage with a booster at a suitable interval with annual boosters thereafter, in order to improve herd immunity. This could interrupt rabies transmission by both urban and sylvatic cycles. By this way vaccination could take the lead role in eradicating rabies from Sri Lanka.

## Conclusion

A single dose of anti-rabies vaccination is not sufficient for the maintenance of antibody titres for a period of 1 year in 40.42% of canines in group B. Median antibody titres of dogs in groups C and D indicate immune responsiveness in animals in group C with previous vaccination history is higher than the animals in group D without vaccination history. Irrespective of whether previously vaccinated dogs in groups E and F were regularly vaccinated or not, antibody titres were above the protective level in most of the dogs on day 60, 180 and 360. Maternal antibodies do not provide adequate protection to puppies in group G (puppies from previously vaccinated dams) until the first anti-rabies vaccination. Immunity development after vaccination seems to be closely similar in both the groups of puppies. There seem to be many factors which affect development of antibodies following vaccination against rabies. Based on these findings, we recommend that puppies should be given two anti-rabies vaccines in the first year of life at suitable time intervals with annual revaccinations. It is necessary to conduct further studies to determine the exact time interval for the first booster vaccine for puppies.

## Abbreviations

ABC: Animal birth control
ARV: Anti-rabies vaccination
IQR: Inter quartile ranges
MOH: Medical officer of health
MRI: Medical research institute
PET: Post exposure treatment
RFFIT: Rapid fluorescent focus inhibition test
WHO: World health organization
